# Supplementary material for: TGFβ and Hippo Signaling Pathways Coordinate to Promote Acinar to Ductal Metaplasia in Human Pancreas
Source: Cells. 2024 Jan 18;13(2):186. doi: 10.3390/cells13020186 (PMC10813953; doi:10.3390/cells13020186)
Supplement: Supplementary file 1 [file cells-13-00186-s001.zip › cells-2730262-supplementary/Supplementary Material Figures.pdf]

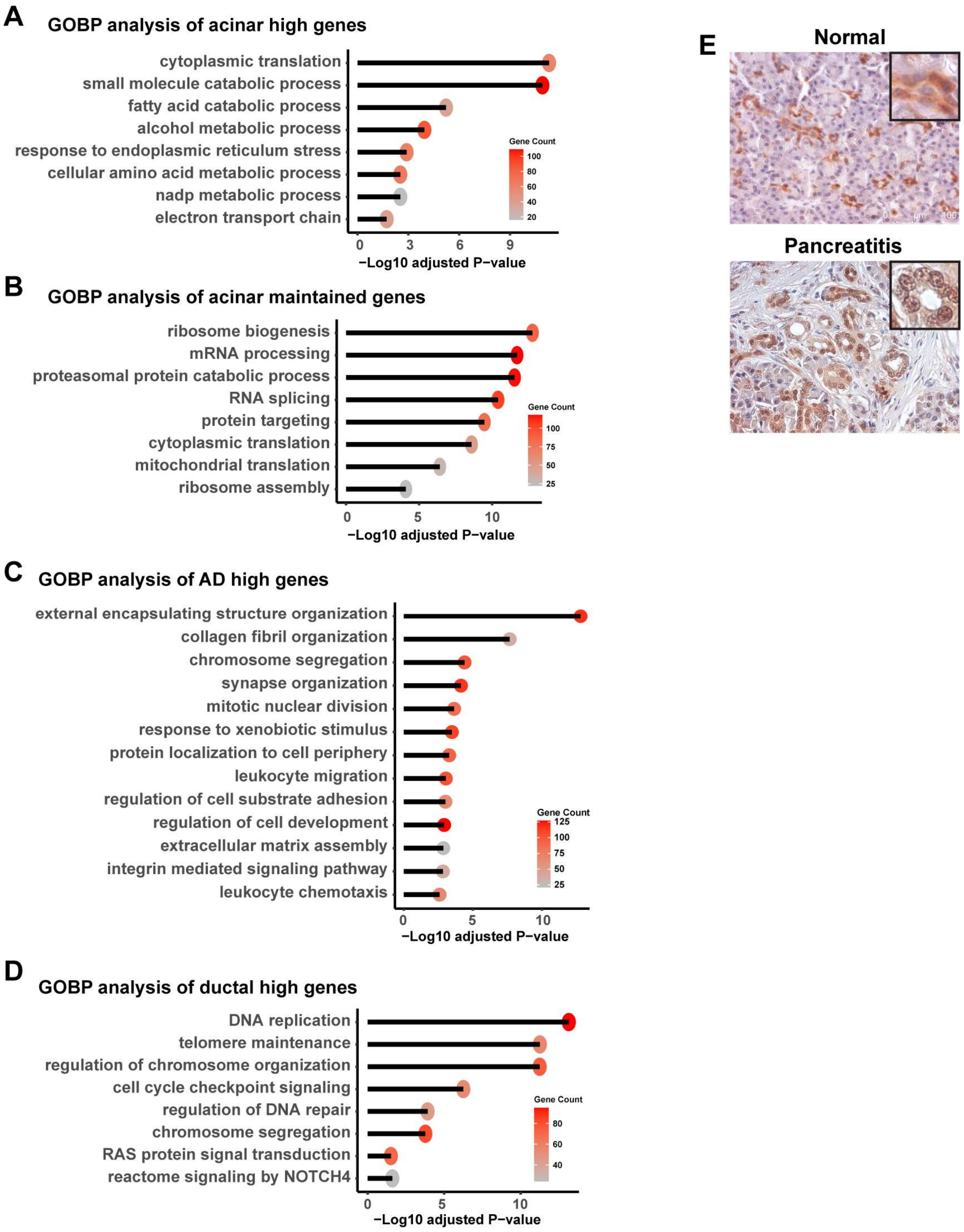

Supplementary Figure S1. Unique transcription profile of human primary acinar cells during ADM

A. Gene ontology of genes highly expressed in acinar cells which are lost during ADM. B. Gene ontology of genes which are highly expressed in acinar cells and maintained during ADM. C. Gene ontology of genes which are not expressed in fresh acinar or ductal cells, but induced in acinar cells during ADM. D. Gene ontology of genes highly expressed in ductal cells but not in acinar or AD cells. E. IHC staining of YAP1 in ductal cells of the normal pancreas (top, insert) and both acinar and ductal cells in pancreatitis (bottom, insert).

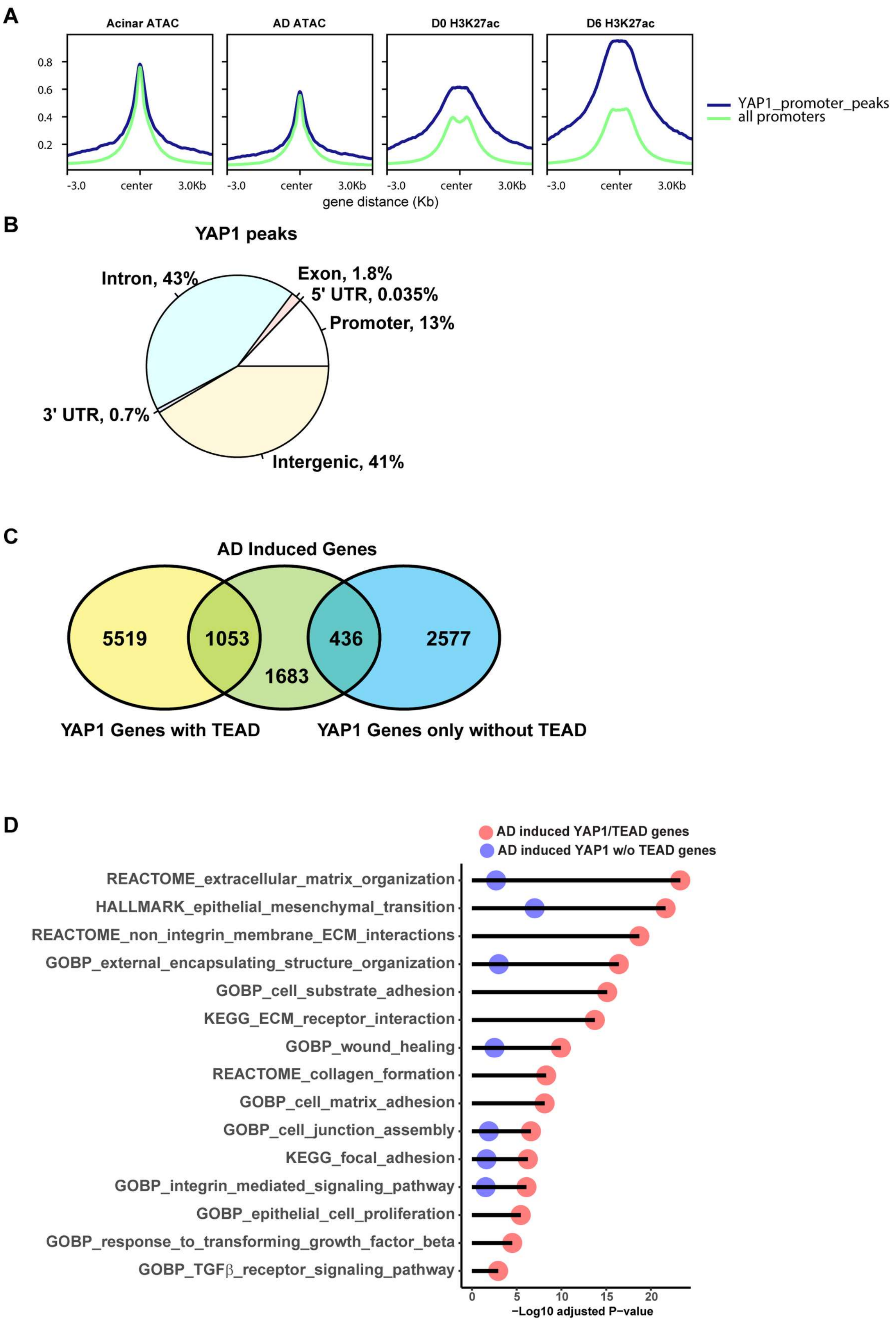

Supplementary Figure S2. YAP1 binds to accessible chromatin regions to induce ADM associated transcriptional programs.

A. Histograms showing ATAC-seq signal and H3K27ac enrichment at YAP1 promoter peaks and all promoter regions in fresh acinar cells and AD cells. B. Pie chart showing proportion of YAP1 peaks

which were annotated to indicated genomic regions. C. Venn diagram showing overlap between AD induced genes and YAP1 bound genes with and without a TEAD binding motif. D. Gene ontology of AD induced genes with YAP1 ChIP peaks with (red) and without (blue) a TEAD binding site.

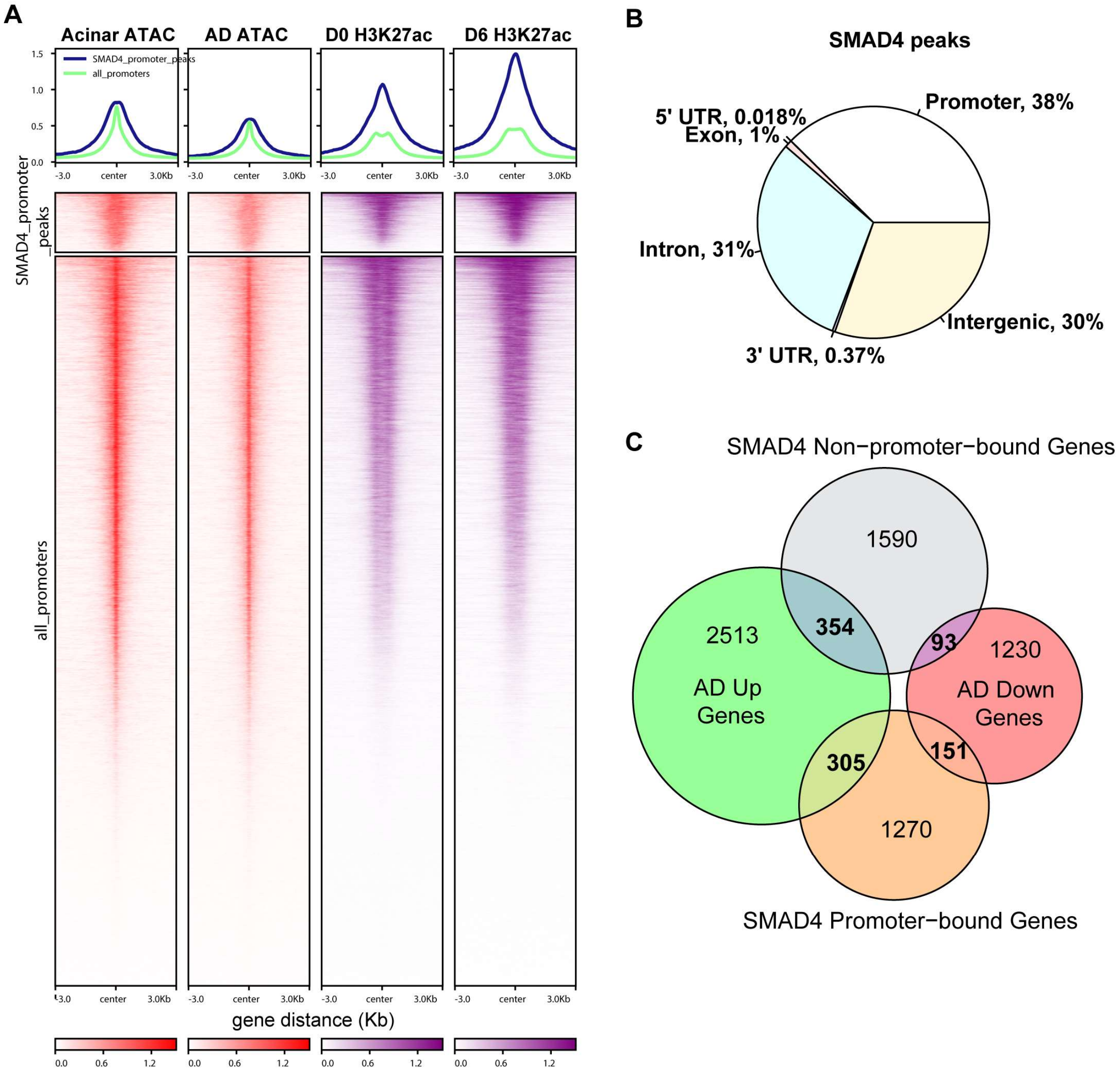

Supplementary Figure S3. SMAD4 and YAP cooperate to induce ADM associated transcriptional changes

A. Heatmaps showing ATAC-seq signal and H3K27ac enrichment at SMAD4 promoter peaks and all promoter regions in fresh acinar cells and AD cells. B. Pie chart showing proportion of SMAD4 peaks which were annotated to indicated genomic regions. C. Venn diagram showing overlapping among AD induced genes, AD repressed genes, SMAD4 promoter-bound genes and SMAD4 non-promoter-bound genes.

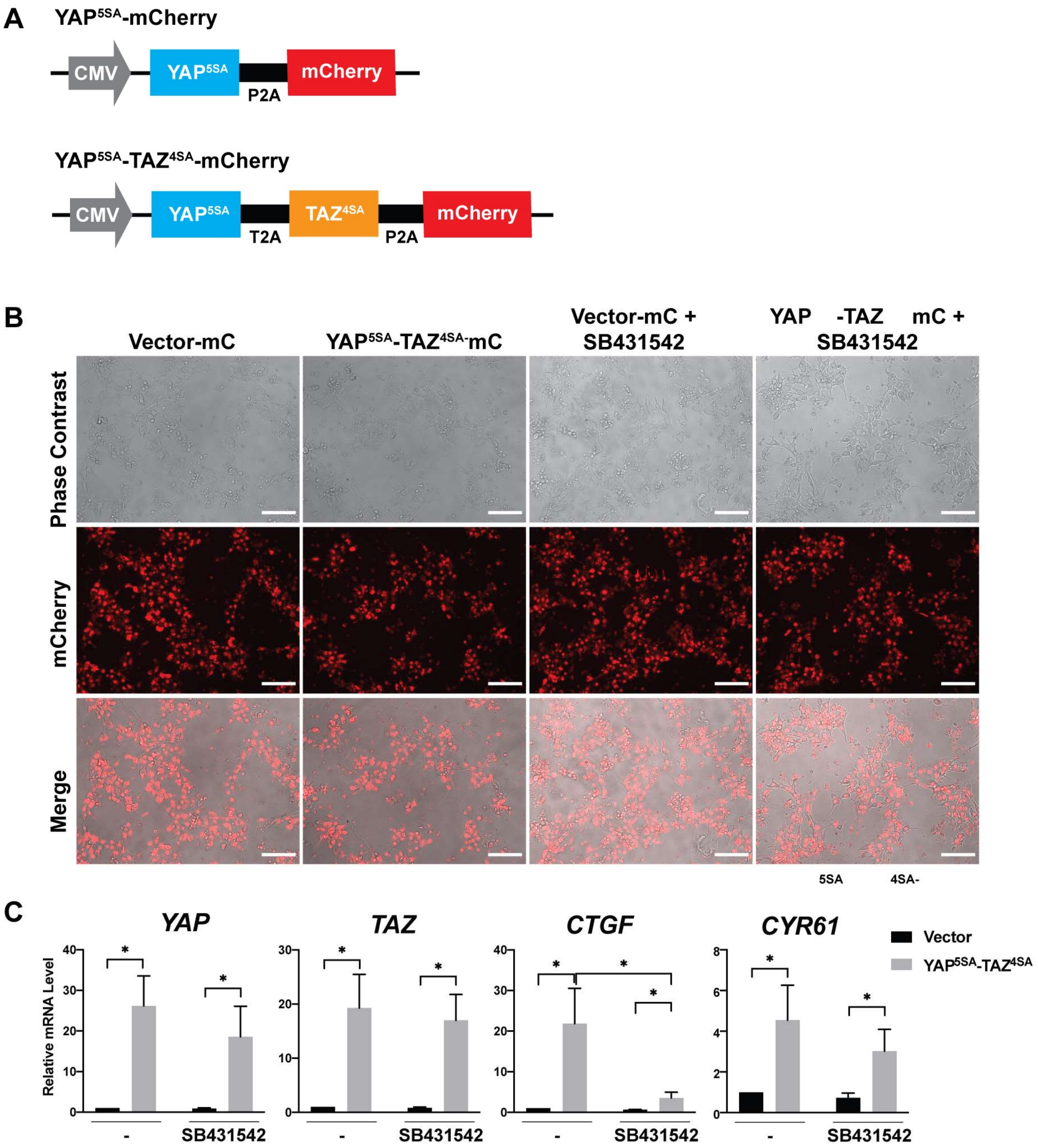

Supplementary Figure S4. YAP1/TAZ overexpression induces the expression of CTGF and CYR61 A. Schematic of lentiviral constructs used for induction of YAP1 and TAZ overexpression. B. Representative images of lentiviruses infection of primary pancreatic acinar cells. Scale bar, 100 μm. C. qRT-PCR analysis showing the expression levels of YAP, TAZ and their targets in acinar cells under different treatment conditions. N=4. Error bars=S.E.M. * $p<0.05$.

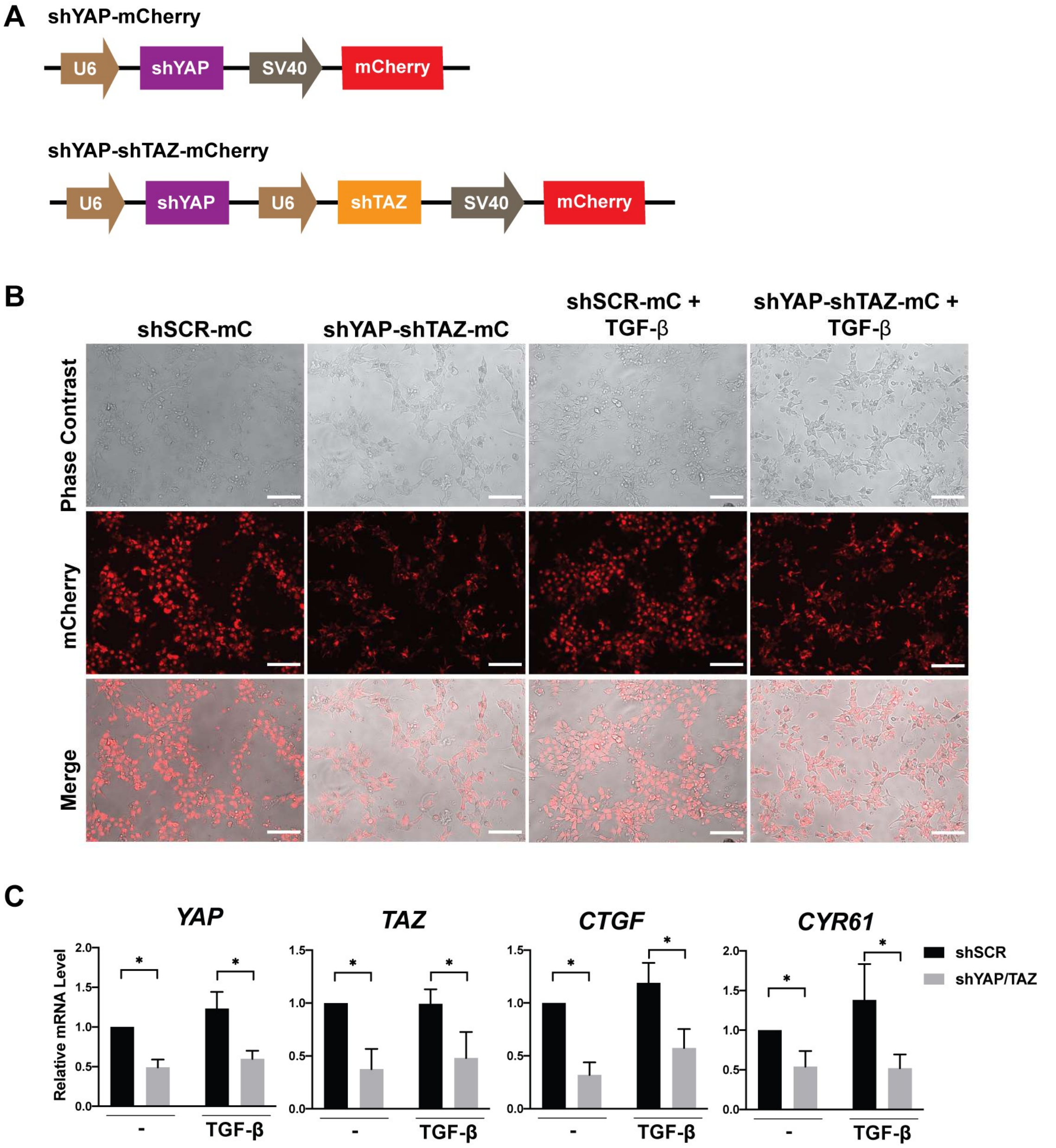

Supplementary Figure S5. YAP1/TAZ knockdown suppresses the expression of CTGF and CYR61. A. Schematic of lentiviral constructs used for knockdown of YAP1 and TAZ. B. Representative images of lentiviruses infection of primary pancreatic acinar cells. Scale bar, 100 μm. C. qRT-PCR analysis showing the expression levels of YAP, TAZ and their targets in acinar cells under different treatment conditions. N=4. Error bars=S.E.M. * $p<0.05$.

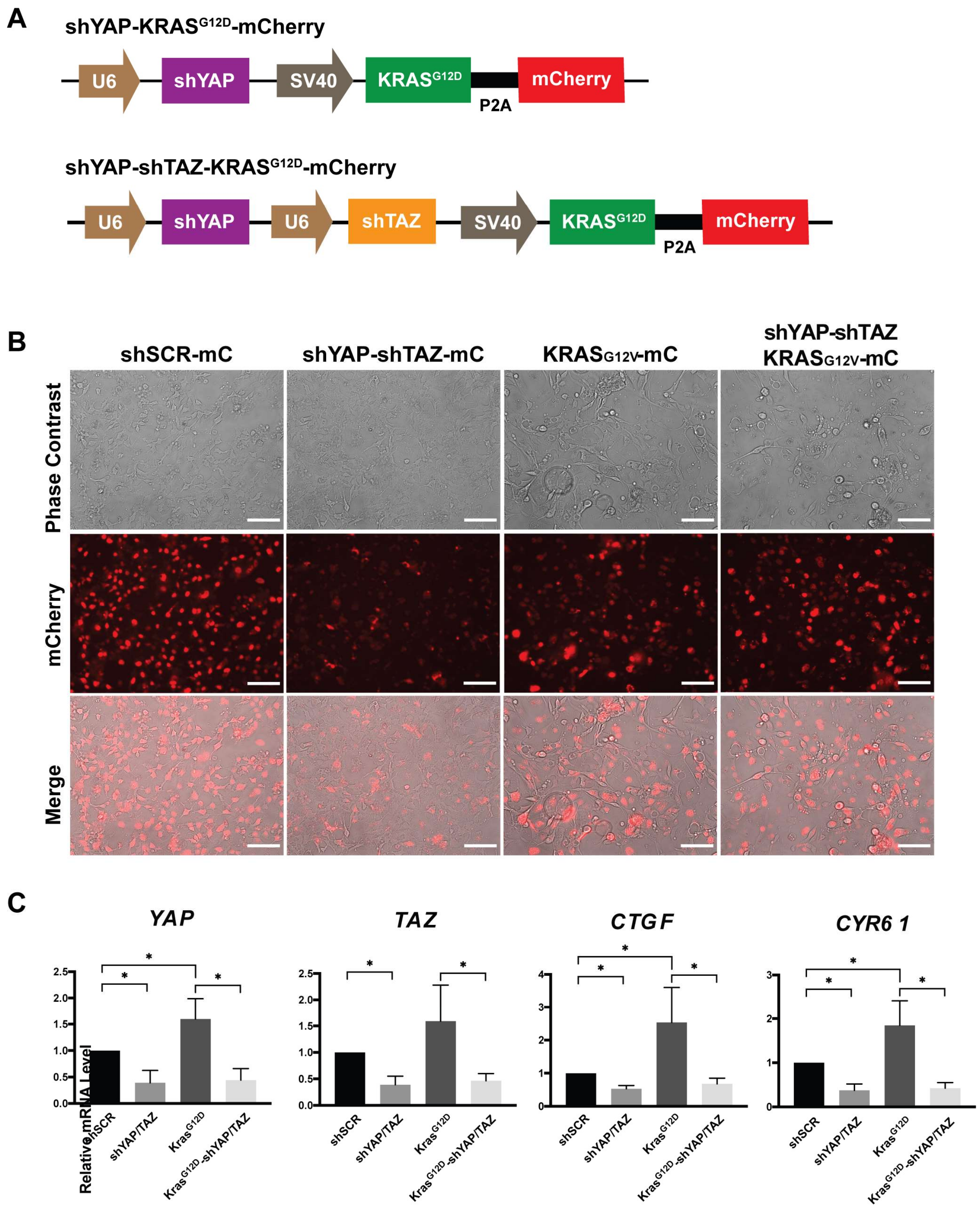

Supplementary Figure S6. YAP1/TAZ knockdown while expressing oncogenic KRAS.
A. Schematic of lentiviral constructs used for induced expression of KRAS-G12D and knockdown of YAP1 and TAZ. B. Representative images of lentiviruses infection of primary pancreatic AD cells. Scale bar, 100 μm. C. qRT-PCR analysis showing the expression levels of YAP, TAZ and their targets in AD cells after infection with different lentiviruses. N=4. Error bars=S.E.M. * $p<0.05$.
